# Regulation of hepatic Sirt1 expression and lipid metabolism through TNF receptor signaling

**DOI:** 10.3389/fimmu.2025.1627433

**Published:** 2025-07-22

**Authors:** Ian N. Hines, Samuel B. Stafford, Blair U. Bradford, Michael D. Wheeler

**Affiliations:** ^1^ Department of Nutrition Science, East Carolina University, Greenville, NC, United States; ^2^ Department of Pharmacology, University of North Carolina, Chapel Hill, NC, United States

**Keywords:** TNFα, non-alcoholic fatty liver, SIRT1, lipid metabolism, fatty liver, choline deficiency model

## Abstract

The production of tumor necrosis factor -alpha (TNFα) has been associated with fatty liver disease (i.e, hepatosteatosis) for many years. In fact, cytokine production has been thought of as a consequence of hepatic lipid accumulation which then becomes a critical factor in the development of chronic liver pathologies as well as in the pathogenesis of insulin resistance. The purpose of this study was to test the hypothesis that TNFα directly regulated lipid metabolism in liver. Wild type mice and mice lacking the receptor for TNFα (TNFR1-/-) were fed control diet or a choline-deficient diet. In addition to pro-inflammatory response, choline-deficient diet increased hepatic lipid accumulation and liver injury, serum triglyceride and insulin levels, as well as increased fasting glucose levels in wildtype mice but to a significantly lesser extent in TNFR1-/- mice. Liver perfusion and metabolic cage studies revealed that TNFR1-/- mice exhibited higher rates of lipid oxidation than wildtype mice. Importantly, TNFR1-/- mice have elevated hepatic expression of metabolic and circadian rhythm regulator SIRT1 in comparison to wild type mice. In isolated hepatocytes, TNF suppressed sirt1 expression while inducing expression of DBC1, a known inhibitor of sirt1 function and expression. These data suggest that TNFα and possibly other innate immune factors play a critical role in the development of hepatosteatosis and the onset of metabolic syndrome. This data also suggests an interplay among innate immunity, hepatic metabolism, and circadian rhythm in the pathogenesis of metabolic syndrome.

## Introduction

Over the past decade it has become clear that inflammation is a key component and perhaps a driving force behind metabolic syndrome, type II diabetes and obesity (1–4). TNF was some time ago discovered as a macrophage-derived factor causing cachexia (5–7). Tumor necrosis factor (TNFα) expression continues to be a clear link between innate immunity and obesity and metabolism (8, 9). To this point, mice lacking the TNF receptor 1 are resistant to diet induced insulin resistance (10–12)).

Diet-induced fatty liver, which is classified as non-alcoholic fatty liver disease, and associated steatohepatitis represent a growing problem clinically, affecting a significant percentage of the US population (13, 14). While it is still not fully clear how inflammatory cytokines impact hepatic metabolism, a growing body of experimental data would also implicate certain pro-inflammatory cytokines as potential initiators and propagators of non-alcoholic steatohepatitis (NASH) (15, 16). The accumulation of lipid associated with NASH represents an important risk factor for several secondary liver pathologies which are often characterized by an early influx of inflammatory cells. Indeed, lipid accumulation depletes antioxidants, increases oxidant stress through lipid peroxidation, both of which may promote DNA damage and hepatic carcinogenesis. Increased lipid accumulation promotes hepatocyte dysfunction, hepatocellular oxidant stress, and hepatic innate immune response activation.

In alcoholic liver disease, Kupffer cell activation and TNFα production are critical for both alcohol-associated hepatocellular injury and tissue lipid accumulation. It is still unclear how immune cell activation and TNFα production are responsible for the development and progression of hepatosteatosis. It was shown by Karin and others that JNK-IKK pathway in hepatocytes is essential for obesity-induced insulin resistance presumably via production of pro-inflammatory mediators (17). It has been demonstrated that liver resident macrophages (i.e., Kupffer cells) were responsible for TNF and chemokine production and the consequential influx of blood monocytes and neutrophils in diet induced steatohepatitis (18, 19). Others have also reported this observation with a focus on macrophage recruitment in the development of hepatic steatosis (20–22).

Despite a clear role of TNF and other chemokines to promote diet-induced inflammation, little is known about how TNF signals the metabolic alterations in NASH. Diehl and others demonstrated decreased lipid accumulation in TNFα-deficient mice fed a high calorie diet (23, 24). On the other hand, Schnyder-Candrian et al. demonstrate enhanced hepatosteatosis in TNFα deficient mice fed a high cholesterol diet when compared to similarly treated wild type mice (25, 26). Much work has investigated JNK, a primary downstream target of TNFR1 in hepatocytes, as a regulator of many hepatic lipid accumulation (27–29). An overwhelming body of literature now implicates Sirt1 as a master controller of fatty acid metabolism and energy homeostasis in liver (30–32). In response to multiple nutritional and hormonal signals, SIRT1 is a NAD+ dependent histone deacetylase (HDAC), that inhibits fatty acid synthesis/storage, stimulates fatty acid β-oxidation, and induces gluconeogenesis (33, 34). New data suggest that a reduction in the SIRT1 activity increases the risk of fatty liver in response to dietary fat (35, 36). Moreover, the loss of SIRT1 expression specifically in liver increases hepatic lipid accumulation (37).

To better understand the role of TNF receptor dependent signaling in the development and progression of fatty liver disease and the possible effect of TNFR1 on sirt1 dependent function *in vivo*, a choline-deficient diet-induced model of liver steatosis was used. The results from these studies demonstrate the profound importance of TNFα signaling through TNFα receptor 1 in the promotion of lipid accumulation within the liver. Most importantly, these studies show that hepatic sirt1 expression is regulated by TNFR1 dependent mechanisms *in vivo* and that TNF suppresses Sirt1expression and function in isolated hepatocytes.

## Detailed methods

### Animals and treatment

Male C57Bl/6J wild type mice or TNFα receptor 1-deficient (TNFR1^-/-^; C57Bl/6- *Tnfrsf1a^tm1Imx^/J*) mice were purchased from Jackson laboratories (Bar Harbor, ME). The genotypes of all animals were verified by standard PCR procedures using primer sequences from the suppliers. Each strain was maintained through established breeding protocols and kept within AAALAC approved facilities and guidelines. The procedures for the care and treatment of mice were followed according to those set by East Carolina University Institutional Animal Care and Use Committee guidelines.

Male wild type or TNFR1^-/-^ mice were fed either standard lab diet or a diet specifically deficient in choline (Dyets, Bethlehem, PA) for a period of 10 weeks. Following feeding, the mice were sacrificed and serum and tissue collected for further analysis of routine parameters of liver injury and lipid accumulation.

### Live animal monitoring

Mice fed regular chow diet were assessed for 48 hours in the TSE LabMaster animal metabolism monitoring system (TSE Systems, Chesterfield MO). Mice were housed for three days to acclimate prior to any measurement. Measurements included oxygen consumption (V_O2_), carbon dioxide production (V_CO2_), respiratory exchange ratio (RER, calculated), calculated heat production, total horizontal ambulatory activity and food and water consumption. NMR-MRI (EchoMRI, Houston TX) analyses were performed for body composition measurements of fat, lean, free water and total water masses in live mice. Results were averaged by photoperiod and analyzed by two-way ANOVA.

### Liver injury parameters

Serum levels of alanine aminotransferase, aspartate aminotransferase, and alkaline phosphatase will be measured by spectrophotometric analysis. Further, a portion of the liver will be fixed in 10% buffered formalin for 24 hours. Tissue will then be embedded in paraffin and sectioned 7μm thick. Sections will then be subjected to routine hematoxylin and eosin staining. Some sections will also be stained for collagen content using picrosirius red stain or Masson’s Trichrome stain using established protocols in the laboratory.

### Liver perfusion

Details of the liver perfusion technique have been described elsewhere (38). Briefly, livers were perfused with Krebs-Henseleit bicarbonate buffer (pH 7.4, 37°C) saturated with an oxygen/carbon dioxide mixture (95:5) in a non-re-circulating system. Perfusate was delivered at flow rates of approximately 4 ml/g liver weight/min via a cannula inserted in the portal vein. Perfusate exited the liver via a cannula placed in the inferior vena cava and was channeled past a Teflon-shielded, Clark-type oxygen electrode. Oxygen uptake was calculated from influent minus effluent oxygen concentration differences, the flow rate, and wet tissue weight. In experiments using livers from fed animals, samples of effluent perfusate were collected and analyzed for glucose, pyruvate, lactate, β-hydroxybutyrate, and acetoacetate by standard enzymatic techniques. In experiments with livers from fasted animals, only β-hydroxybutyrate and acetoacetate were measured

### Histopathology and immunohistochemistry

Tissue was fixed in 4% paraformaldehyde for 24 hours and subsequently embedded in paraffin. Tissue sections were prepared (7mm thick) and stained with routine hematoxylin and eosin. Immunohistochemistry for CD3 and F4/80 were performed as previously described (39, 40).

### Real time reverse transcriptase polymerase chain reaction

Total RNA from liver was isolated using the Trizol reagent (Gibco/ThermoFisher Scientific, Grand Island NY) according to the manufacture’s recommendations. Total RNA was used to synthesize cDNA using. For quantification of message expression, cDNA was amplified using primer sequences in the presence of Sybr Green (Applied Biosystems/ThermoFisher Scientific, Grand Island NY) using a standard PCR protocol (95°C for 10s, 57°C for 15s, and 72°C for 20s, total of 40 cycles. B-actin and/or 18S message expression was used as the house keeping gene and for quantification of relative expression levels using the comparative method of quantification.

### Electromobility shift assay

Total liver cytosolic and nuclear protein will be isolated. Protein concentrations will then be quantified immediately using a Bio-Rad Protein Assay kit according to the manufacturer’s instructions. Extract will be incubated in the presence of P32-labelled oligo probes for 30 min. Free probe will be resolved from bound probe using 19:1 acrylamide:bisacrylamide gel electrophoresis. Labeled probe will be visualized on film be autoradiography. DNA binding intensity was determined using Image J densitometry (https://imagej/ij/).

### Western blotting

Total liver cytosolic and nuclear protein will be isolated. Protein concentrations will then be quantified immediately using a Biorad Protein Assay kit according to the manufacturer’s instructions. Proteins (50 μg for cytosolic or 10 μg for nuclear) will be diluted in 2X Lamaelle buffer to a volume of 30 μl and then separated on 4-16% Tris-glycine acrylamide ClearPage gels (CBS Scientific, San Diego CA). Proteins will then be transferred to nitrocellulose membranes and subsequently blocked for 12 hours with non-fat dry milk (5%) dissolved in tris-buffered saline containing 0.1% Tween. Membranes will be incubated antibody in T-TBS+5%NFDM for 16 hours at 4°C. After thorough washing in T-TBS, membranes will be incubated with secondary antibodies conjugated to horse radish peroxidase at a concentration of 1:1000 for 1 hour at room temperature. After thorough washing, labeled proteins will be visualized on film with enhanced chemiluminescence (Thermo Scientific, Grand Island NY).

### Hepatoctye isolation and culture

Hepatocytes from fed and fasted rats were isolated by standard techniques described elsewhere (41). Briefly, livers were perfused with a Krebs-Ringer-HEPES buffer containing 0.2% collagenase (Sigma-Aldrich, St Louis MO). Livers were isolated and cells were dispersed by gentle shaking and filtered through sterile nylon gauze. The cells were washed two times with sterile phosphate-buffered saline and then purified by centrifugation in 50% isotonic Percoll (Sigma-Aldrich). Cells were resuspended with Krebs-Ringer-HEPES + Ca^2+^ buffer to a total volume of 10 ml. Viability was validated via trypan blue exclusion and routinely exceeded 90%.

### Statistics

All data will be expressed as mean SEM (standard error of mean) for at least 6-8 animals per group. Statistical significance among groups was be determined 2-way ANOVA followed by Bonferoni’s *post-hoc* analysis to determine within-group differences. In some studies, student’s t test was used for statistical analysis. Significance was be set at p<0.05.

## Results

### Loss of TNFR1 blunts experimental induced fatty liver disease in mice

To address the role of TNFα in the development and progression of fatty liver disease, the choline-deficient diet models of liver steatosis was used. The link between choline deficient and fatty liver has long been recognized and recently examined in humans. (42, 43). Choline-deficient diet is a well-established model of hepatosteatosis, generating timely and severe hepatocellular fat accumulation with mild inflammation. The results from these studies demonstrate the profound importance of TNFα signaling through TNFα receptor 1 in the promotion of lipid accumulation within the liver using this model.

To test the hypothesis that TNFα was critical for the development of NAFLD caused by choline-deficient diet, wild type and TNFR1-/- mice were given standard AIN-76 (fully supplemented) diet or L-amino acid defined choline-deficient AIN-76 diet for 4 and 10 weeks. At 4 or 10 weeks, mice were sacrificed; serum, liver, spleen, muscle and adipose was harvested.

**Figure 1:**
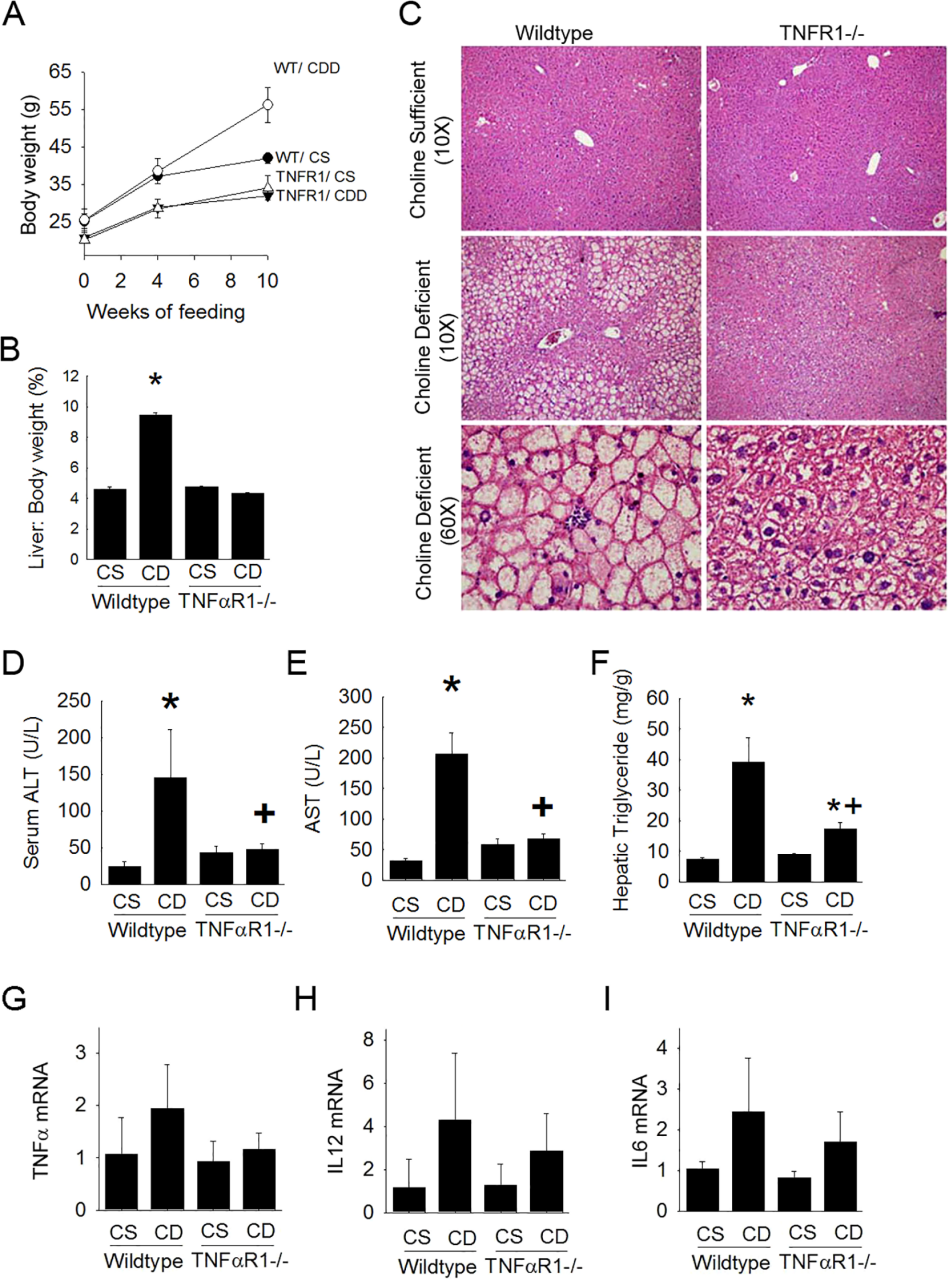
TNFR1-/- mice are resistant to experimental fatty liver disease. Wild type and TNFR1-/- (22-25 g) were fed ad libitum control (Choline sufficient, CS) diet or diet deficient in choline (Choline Deficient, CD) for ten weeks. **(A)** Body weight changes at 4 and 10 weeks of diet. **(B)** Liver to body weight ratio (% of body weight) after 10 weeks of diet. **(C)** Representative liver histology (H&E) is shown at 10X and 60X. **(D)** Serum levels of ALT after 10 weeks of diet. **(E)** Serum levels AST after ten weeks of diet. **(F)** Hepatic triglyceride levels were measured in liver lysates of animals fed diet for 10 weeks. **(G–I)** Hepatic mRNA levels for pro-inflammatory cytokines TNF, IL12 and IL6 were measured by quantitative PCR. Data are representative of 4-6 animals per group and are expressed as mean SEM. *p<0.05, compared to wildtype fed CS diet. Two-way ANOVA with Bonferoni *post-hoc* analysis was performed.

Choline-deficient diet induced time-dependent weight gain and hepatosteatosis in wild type mice becoming apparent within 4 weeks and becoming more severe over 10 weeks (**Figures 1A–C**). However, livers from TNFR1^-/-^ mice fed choline-deficient diet were resistant to the development of fatty liver. Furthermore, evaluation of liver weight to body weight ratios supported the histopathological assessment of lipid accumulation. Wild type mice presented with large increases in LW/BW following 10 weeks of CDD feeding when compared to their CSD-fed controls. Absence of TNFαR1 significantly blunted the CDD-induced increase in LW/BW ratio.

In wildtype mice ALT levels and AST levels were elevated with choline-deficient diet after 10 weeks (**Figures 1D, E**). Interestingly, these increases were nearly completely blunted in the TNFR1-/- mice on choline-deficient diet. Hepatic triglyceride content was also measured biochemically (**Figure 1F**). Consistent with other parameters of hepatic lipid accumulation, hepatic triglycerides were significantly increased nearly 8 fold in wild type mice fed choline-deficient diet compared to that of control fed animals. This increase was largely blunted in livers of TNFR1-/- mice.

Hepatic pro-inflammatory cytokine expression associated with hepatic steatosis was assessed (**Figures 1G–I**). TNFα, IL12, and IL6 expression was elevated in wildtype mice fed choline-deficient diet. This increase, although not statistically different, was blunted in TNFR1-/- mice fed choline-deficient diet.

### Chronic experimental induced fatty liver disease promotes metabolic dysfunction which is dependent upon TNFR1 expression

**Figure 2:**
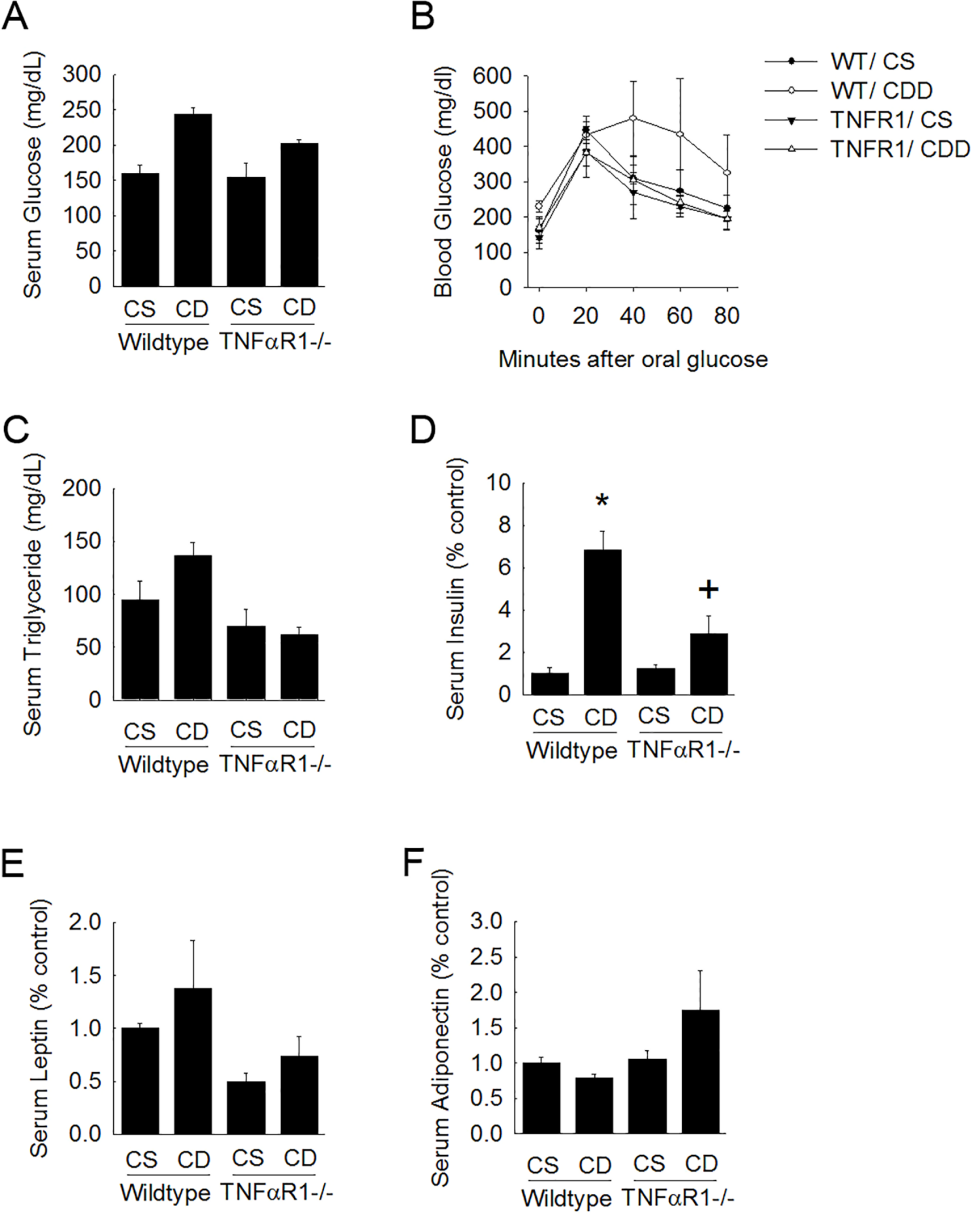
TNFR1-/- mice are protected from metabolic changes associated with fatty liver disease. Wild type and TNFR1-/- (22-25 g) were fed ad libitum control (Choline sufficient, CS) diet or diet deficient in choline (Choline Deficient, CD) for ten weeks. **(A)** Fasted serum glucose was measured. **(B)** Blood glucose levels were measured after oral glucose challenge in animals fed control or choline deficient diet for 10 weeks. **(C–F)** Serum levels of triglycerides, insulin, leptin, and adiponectin were measured in animals after 10 weeks of diet. *p<0.05, compared to wildtype fed CS diet; ^+^p<0.05, compared to wildtype fed CD diet. Two-way ANOVA with Bonferoni *post-hoc* analysis was performed.

Wild type mice on choline-deficient diet weighed 60 ± 4 g compared to control fed mice which were within normal age-matched range for C57Bl6 (34 ± 6 g). Body weight gain caused by CDD was not observed in TNFR1-/- mice. After 10 weeks of choline-deficient diet, several metabolic factors (i.e, fasting serum glucose, OGTT, serum triglycerides, insulin, leptin and adiponectin) were measured (**Figure 2**). Blood glucose concentration following a 2.5g/kg oral gavage of glucose after an overnight fast was measured in both wild type mice and TNFR1-/- mice at 10 weeks of choline-deficient diet. Fasting blood glucose was elevated in wildtype CDD-fed animals (**Figure 2A**). This increase was blunted in TNFR1-/- mice fed CDD diet. Moreover, glucose clearance after an oral glucose challenge was much more rapid in TNFR1-/- mice compared to that of the wild type mice (**Figure 2B**). serum triglycerides were significantly elevated after 10 weeks of CDD. Serum triglyceride levels as well as serum leptin levels were increase slightly in wildtype mice fed CDD diet. This increase was not observed in TNFR1-/-mice (**Figures 2C, E**). Importantly, serum insulin was elevated to nearly 8-fold in CDD-fed mice, compared to wildtype control-fed mice. This increase in serum insulin was significantly blunted in TNFR1-/-. While many of these measures were not statistically significant, these data do suggest that choline-deficient diet can produce features consistent with the development of metabolic syndrome in mice at 10 weeks. It is reasonable to hypothesize that longer exposure to CDD would produce more significant changes regarding these metabolic features. Most importantly, these data suggest that TNFα might play a larger role in the pathogenesis of metabolic syndrome that is initiated in this unique model.

### TNFR1 mice have elevated hepatic lipid metabolism

**Figure 3:**
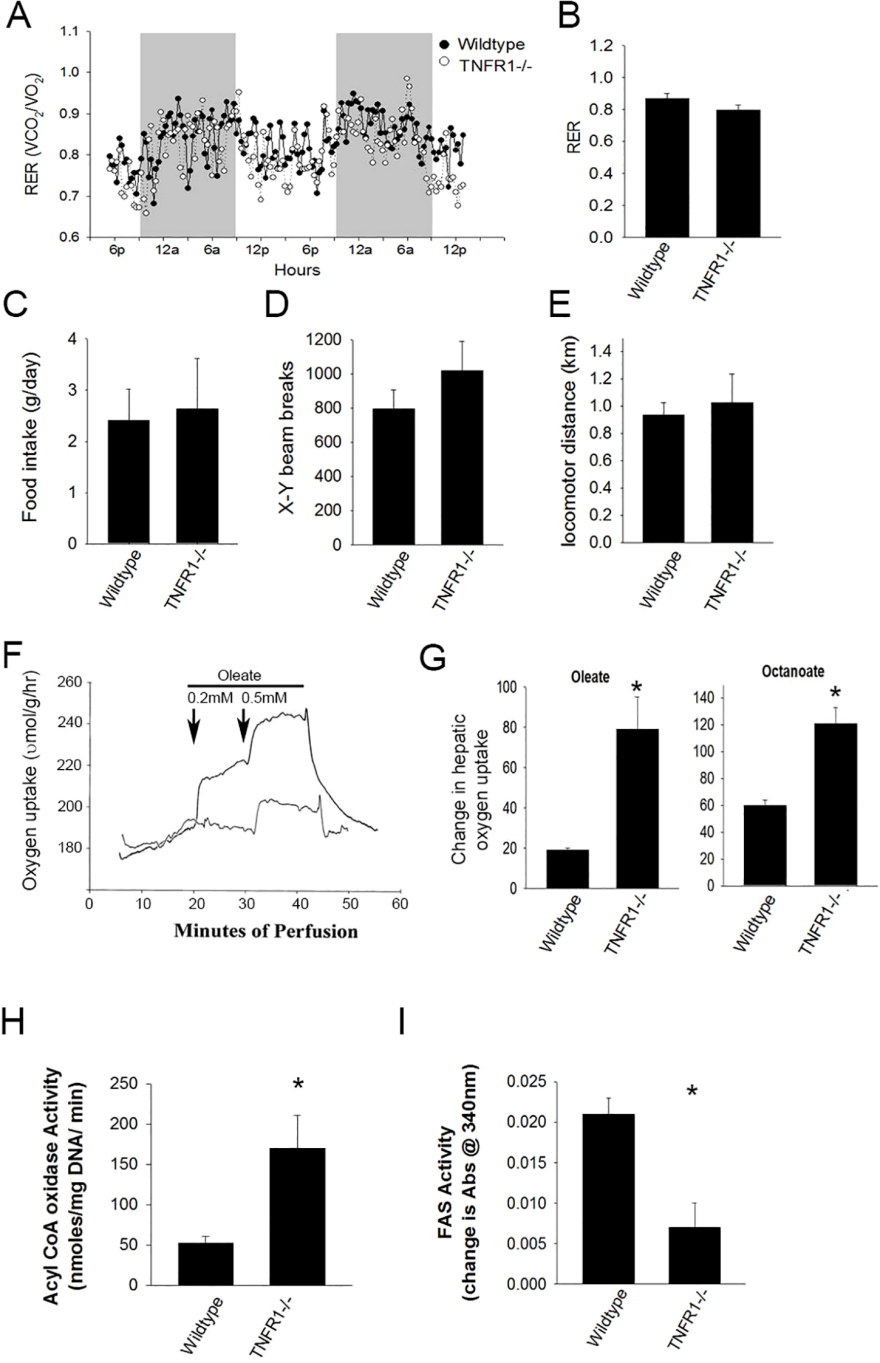
Loss of TNF receptor alters energy utilization. Wild type and TNFR1-/- (22-25 g) were on control diet at 10 weeks of age were housed in metabolic chambers for 2 days for monitoring. **(A)** Representative data is shown for calculated respiratory exchange ratio (RER) for wildtype and TNFR1-/- mice. **(B)** Average RER over 48 hours for wildtype and TNFR1-/- mice. **(C–E)** Food consumption, average x-y beam breaks, and average total distance locomotor activity for wildtype and TNFR1-/- mice. **(F)** Livers from wild type and TNFR1-/- mice fed control diet for 10 weeks were perfused with buffer containing oleate (0.2-0.5 mM) and octanoate (not shown). **(G)** Change in oxygen concentration as a result of oleate or octanoate perfusion was measured. **(H, I)** Acyl CoA oxidase and fatty acid synthase activity in liver lysates from wildtype and TNFR1-/- mice was measured biochemically. Data are representative of four animals per group and are expressed as mean SEM. *p<0.05, compared to wildtype mice. Simple Student’s t-test was performed.

To investigate the role of TNF in metabolic regulation, control fed wildtype and TNFR1-/- mice were housed in metabolic chambers for measuring food and water intake, locomotor activity, O_2_ consumption and CO_2_ production (**Figure 3**). Oxygen consumption and CO_2_ production was measured and used to calculate the respiratory exchange (RER) in both wildtype and TNFR1-/- mice. In wildtype mice, the changes in RER followed a cyclic pattern coinciding with nocturnal activity but averaged 0.86 ± 0.03 over the 48 hour test period (**Figures 3A, B**). This is consistent with published measurements for this strain. In TNFR1-/- the cyclic pattern of the average RER over the 48 hours is 0.79 ± 0.03, which is indicative of increased lipid utilization as an energy source. Neither food intake or water consumption was statistically different between the strains. Locomotor activity assessed by x-y-z light beam breaks in wildtype mice was consistent was published reports. There was a slight increase in activity in TNFR1-/- mice housed under these standard 12-hour light-dark cycles.

### TNFα suppresses hepatic fatty acid oxidation

To explore the hypothesis that TNFR1 dependent signaling regulated hepatic fatty acid oxidation, livers isolated from wild type and TNFR1-/- mice were isolated and perfused with Ringer-balanced salt solution containing either 0.2 or 0.5 mM oleate, a medium chain fatty acid requiring CPT1-dependent transport into the mitochondria and octanoate, a short chain fatty acid which is metabolized independent of CPT1 transport. CO_2_ (as an inverse of O_2_ consumption) was measured in the perfusate (**Figure 3F**). Interestingly, metabolism of both oleate and octanoate was significantly increased in TNFR1-/-, compared to wild type mouse liver, suggesting that the increase in fatty acid metabolism was likely due to increased oxidation (**Figure 3G**). Further, Acyl CoA oxidase activity and Fatty acid synthase activity was elevated in TNFR1-/- liver extract compared to wildtype (**Figures 3H, I**). These data suggest clearly that TNFα plays a regulatory role in the metabolism of hepatic fatty acids.

### Basal SIRT1 gene expression is increased in the absence of TNF signaling *in vivo*

**Figure 4:**
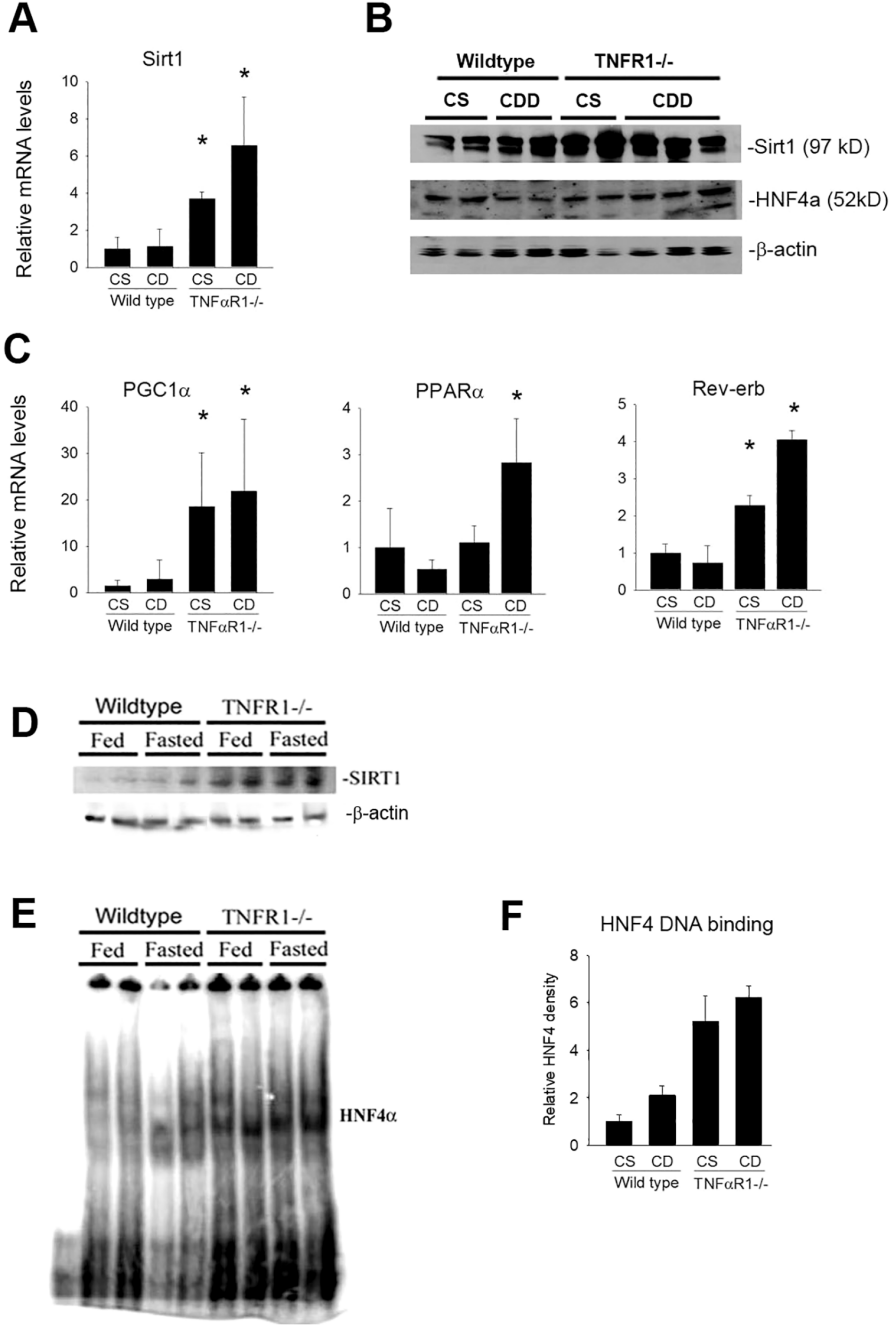
Hepatic SIRT1 expression is increased in the absence of TNF receptor signaling. Wild type and TNFR1-/- (22-25 g) were fed ad libitum control (Choline sufficient, CS) diet or diet deficient in choline (Choline Deficient, CD) for ten weeks. **(A)** Hepatic mRNA levels for Sirt1 after 10 weeks of diet. **(B)** Western blot for SIRT1, HNF4α and β-actin using extract from wildtype and TNFR1-/- livers fed control and CD diet for 10 weeks. **(C)** Hepatic mRNA levels for PGC1α, Rev-erb and PPARα after 10 weeks of diet. **(D)** Wild type and TNFR1-/- (22-25 g) were fed ad libitum control diet (Fed) or fasted overnight (Fasted). Representative Western blot for SIRT1 using liver extract. **(E)** Representative electromobility shift assay for HNF4*a* using hepatic nuclear extracts from wildtype and TNFR1-/- mice fed control diet or fasted overnight. **(F)** HNF4a DNA binding activity quantified using Image J image densitometry. Data are representative of four animals per group and are expressed as mean SEM. *p<0.05, compared to wildtype mice.

To assess the mechanism of TNFα suppression of fatty acid metabolism, the effect of TNFα on fasting SIRT expression was evaluated (**Figure 4**). SIRT1 is a major stress-sensing regulator linked to a number of cellular responses such as metabolism, growth and survival (44). Sirt1-dependent regulation of energy homeostasis involving PGC1α, HNF4 and PPARα has been described. Sirt1 gene expression was measured in livers of wildtype mice on control and choline-deficient diet (**Figure 4A**). After 10 weeks of CDD diet, hepatic Sirt1 expression not significantly changed in wildtype animals. Importantly, increases in Sirt1 mRNA levels were observed in livers TNFR1-/- mice. Notably, TNFR1-/- mice fed control diet had a significant increase in the basal expression of Sirt1 compared to wildtype mice. The increase was slightly greater in TNFR1-/1 mice fed CDD diet compared to TNFR1-/- mice fed control diet. Protein levels of Sirt1 and HNF4a were also assessed by Western blot analysis (**Figure 4B**). Changes in protein levels in wildtype and TNFR1-/- after CDD diet were not remarkable.

It is important to note that SIRT expression is not indicative of its deacetylase activity, thus the hepatic expression of lipid metabolism genes known to be regulated by SIRT1 were assessed (**Figure 4C**). While not direct evidence of TNFα regulation of SIRT1 activity, the data do suggest a role for TNFα to regulate expression Sirt1 and several down-stream targets including PGC1α, PPARα and Rev-erb within liver.

Sirt1 can regulate gene expression through forkhead transcription factors, including HNF4α (45, 46). HNF4α DNA binding activity was directly assessed. Since fasting strongly induces SIRT1 as well as HNFa activity, wildtype and TNFR1-/- mice were fasted overnight. SIRT1 gene expression was modestly increases in wildtype mice by fasting. However, Sirt1expression was significantly increased in TNFR1-/- mice under control conditions as after fasting (**Figure 4D**). Similarly, HNF4a DNA binding was only slightly increased in wildtype mice after fasting (**Figure 4E**). HNF4 activity was significantly increased in TNFR1-/- mice (**Figures 4E, F**). These data are consistent with the hypothesis that TNFα regulates expression of key hepatic metabolism genes and that the TNF-dependent expression changes in key metabolic genes correlates with Sirt1 expression and HNF4α activity.

### TNF suppresses SIRT1 activity in isolated hepatocytes

**Figure 5:**
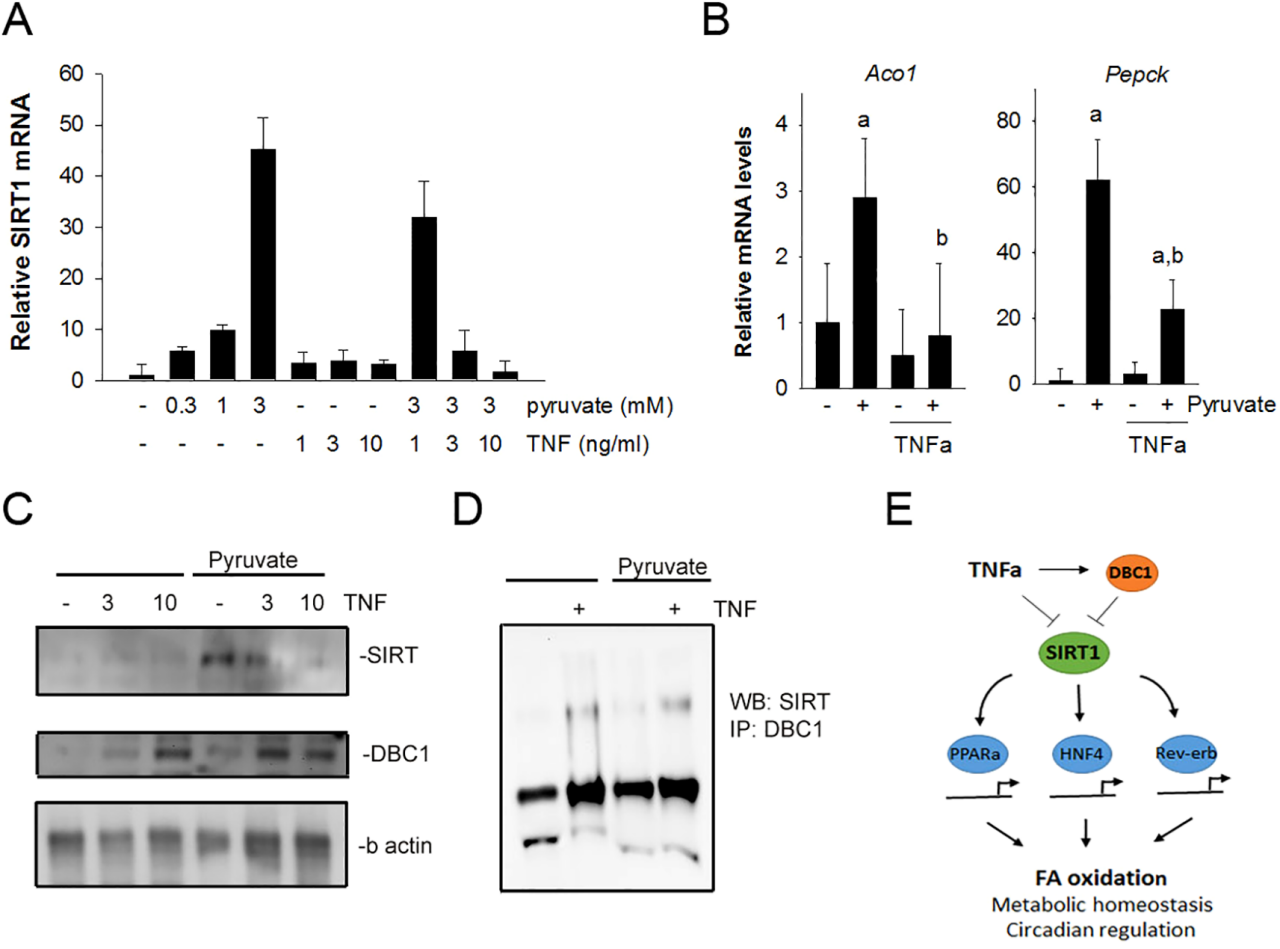
TNFα suppresses Sirt1 expression and increases DBC1 interaction with Sirt1 in isolated hepatocytes. **(A)** Hepatocytes were isolated from wildtype mice and treated with pyruvate (0.1 -10mM) in the absence and presence of TNFα (0.1 -10 ng/mL). After 1 hour, mRNA levels for sirt1 were assessed using qPCR. **(B)** Hepatocytes were treated with 3 mM pyruvate in the absence or presence of 3 ng/mL TNFα. mRNA levels for Aco1 and Pgc1α were assessed using qPCR. **(C)** Hepatocytes were treated with 3 mM pyruvate in the presence or absence TNFα (3 and 10 ng/mL) for 4 hours. Cell lysates were analyzed by Western blot with antibodies against SIRT1, DBC1 and b-actin. **(D)** Lysates from hepatocytes were immuno-precipitated using antibodies against DBC1. Immuno-precipitates were analyzed by Western blot using antibodies against SIRT1. **(E)** Schematic of the working hypothesis for the suppression of SIRT1 function by TNFα through DBC1. TNFα induced suppression of SIRT1 is consistent with a decrease in oxidative metabolism of lipid in hepatocytes.

Since it was demonstrated that Sirt1 expression was increased in the absence of TNFR1, the effect of TNFα on Sirt1 expression and function was directly assessed *in vitro* using primary hepatocytes isolated from wildtype mice. Sirt1 expression was induced *in vitro* by pyruvate at various concentrations. As expected, based on published reports, pyruvate caused a dose-dependent increase in Sirt1 mRNA levels. Hepatocytes were also exposed to recombinant TNFα. TNFα alone had minimal effect on Sirt1 expression in primary hepatocytes; however, TNFα significantly blunted pyruvate-induced Sirt1 expression (**Figure 5A**). These data support the hypothesis that TNF signaling represses Sirt1 expression in hepatoctyes and are consistent with our *in vivo* observations that TNFR1 signaling relates Sirt1 expression and hepatic fatty acid oxidation.

Sirt1 is a potent regulator of hepatic fatty acid oxidation and gluconeogenic pathways. Thus, the expression of acyl CoA oxidase (Aco1) and PPAR coactivator 1α (PGC1a) were assessed in primary hepatocytes (**Figure 5B**). Pyruvate induced expression of both Aco1 and PGC1a. The increase in expression of both were blunted significantly in the presence of recombinant TNFα. These data are consistent with the hypothesis that TNF suppresses hepatic sirt1 expression and the expression of key metabolic regulators downstream of Sirt function. In addition to Sirt1 mRNA changes, Sirt1 protein levels were assessed in isolated hepatocytes under similar conditions as described above (**Figure 5C**). Pyruvate caused an increased in Sirt1 expression that was inhibited by TNFα in a dose-dependent manner.

Recent studies have revealed that cell cycle and apoptosis regulator 2 (CCAR2), also known as Deleted in Breast Cancer 1 (DBC1), is a potent inhibitor of SIRT1 function in hepatocytes (47). Chini et al. reported that DBC1 expression negatively regulates SIRT1 activity in liver via a direct interaction (48, 49). Experiments were done to investigate the relationship between SIRT1 and its potential regulator Deleted in Breast Cancer 1 (DBC1). We show a TNFα-dependent increase in DBC1 expression in hepatocytes both in the absence and presence of pyruvate (**Figure 5C**). To further explore the DBC1 effect on SIRT, co-immunoprecipation experiments were performed to assess the direct interaction between DBC1 and SIRT, which is the reported mechanism of inhibition. A weak, direct DBC1: Sirt1 interaction was observed in hepatocytes treated with TNFα, both in the absence and presence of pyruvate (**Figure 5D**). These data are consistent with previous observations of the DBC1:Sirt1 interaction. Moreover, these data suggest that TNFα promotes DBC1 expression and DBC1 interaction with Sirt1, supporting the hypothesis that TNF suppresses Sirt-dependent function within hepatocytes.

## Discussion

A number of pro-inflammatory cytokines and chemokines including TNF up-regulated in fatty livers (16, 50–54). Hepatic lipid accumulation results in cytokine release, TNFα being the chief, prototypical pro-inflammatory cytokine. Innate immune responses activated within fatty livers have great potential for amplification (17, 50, 55–57). Once cytokine production is initiated, these pro-inflammatory cytokines propel the progression from steatosis to steatohepatitis. Evidence for the participation of TNFα in fatty liver disease is overwhelming, although direct evidence from animal models has been mixed (23, 58–60 *;* 61–65). Specifically how TNFα contributes to hepatic lipid metabolism is a gap in our understanding. TNFα is a potent inflammatory mediator derived from a variety of cell types which interacts with a wide range of signaling pathway. For example, loss of TNFα function improves insulin sensitivity in obese mice, suggesting that TNFα regulates insulin action (10, 66, 67). Importantly, we have demonstrated here and previously in an ethanol-diet model of steatosis/steatohepatitis that TNFα indeed is crucial for the accumulation of lipid in the liver. Here, we used the TNFR1-/- deficient mice to determine its role in the general mechanisms of fatty liver disease caused by choline-deficient diet. Importantly, these data suggest that hepatic inflammation may not be due to the accumulation of lipid in liver but actually may precede and promote the accumulation of lipid, affirming the notion that cytokines drive and potentiate metabolic dysfunction. The data suggest that TNFR1 signaling is important for the early metabolic disarrangement leading to steatosis as well as the pro-inflammatory cascade.

The choline-deficient diet model of non-alcoholic steatohepatitis was used here since it is a liver centric model which blunts the export of triglycerides in very low density lipoprotein (68–71). A surprising outcome of the choline deficient diet, in addition to robust steatohepatitis, was the impact on metabolic homeostasis. It is clear from these experiments that TNF is a critical factor in steatohepatitis, and it is also suggestive that TNF is a contributor to peripheral insulin resistance. This is consistent with the longstanding hypothesis that TNFα is an important player in the crosstalk between liver fat metabolism and peripheral insulin resistance. Choline-deficient diet for 10 weeks resulted in increased blood triglycerides levels, fasting glucose levels and insulin levels in wildtype mice. After 40 weeks, the effects of the choline-deficient diet were even more pronounced and coupled with increased insulin resistance (data not shown). Importantly, TNFR1-/- mice were largely refractory to the chronic metabolic changes associated with the choline deficient diet. What is interesting is that loss of TNFα served to maintain metabolic homeostasis not only in liver but elsewhere. It is intriguing to speculate that since the choline deficient diet model is a liver centric model of lipid accumulation that liver metabolic status is an important physiological feature of peripheral metabolic homeostasis. Clearly, fatty liver disease is strongly linked to obesity and insulin resistance. However, that fatty liver precedes and promotes insulin resistance/and or metabolic syndrome is an interesting observation and one interpretation of the data here would support that notion. It is recognized that HCV infection can induce hepatosteatosis and subsequent insulin resistance (72–74), thus the idea that hepatic metabolic changes are capable of driving peripheral metabolic disease is justified. Circulating levels of TNFα are increased in obesity, type II diabetes and fatty liver disease; TNF clearly impairs insulin signaling. Whether or not hepatic derived TNF is responsible for peripheral metabolic alterations is not clear. TNFα has been demonstrated to promote hyperlipidemia as well as suppress fatty acid oxidation in liver as well as regulate lipid metabolism in several other tissues (75, 76). For example, TNFα inhibits fatty acid oxidation by suppressing acyl CoA oxidase (77, 78). Here, acyl CoA oxidase expression was increased in livers of TNFR1-/- mice.

How TNFα imparts its effects on hepatic fat metabolism is a central question here. It is demonstrated here that, in the absence of TNFR1 signaling, livers more efficient utilize fatty acids as a fuel source, suggesting that TNF suppresses fatty acid oxidation perhaps through the expression of genes involved in multiple metabolic pathways. Here, TNFα suppresses the expression and activity of Sirt1 in hepatocytes, a novel finding that may explain TNFα’s pleotropic effect on energy homeostasis. Sirt is a NAD+ dependent deacetylase that acts as a nutrient/energy sensor. Studies overwhelmingly demonstrate a role of Sirt1 in both hepatic gluconeogenesis and fatty acid metabolism likely through activation of downstream targets such as PGC1α and PPARα (32, 33, 37, 79–82). Loss of sirt1 in liver results in hepatic steatosis, inflammation, impaired PPAR signaling and decreased fatty acid oxidation. Alternatively, Sirt1 activation has been shown to have beneficial metabolic effects in type 2 diabetes. Sirt activation specifically in liver of obese insulin-resistant mice significantly improved fatty liver, normalized hyperglycemia, as well as improved systemic insulin sensitivity (32, 79). It is important to note that expression is only part of the capacity of Sirt1 to act and that Sirt1 deacetylase activity should also be considered. Changes in downstream SIRT targets such as HNF4α, PPAR, PGC1 and Rev-erb are only indicative of SIRT1 activity but are also influenced by other metabolic regulators, including FOX and NFkB transcription factors. Sirt1’s interaction with these other factors can also impact gene expression and cellular responses associated with cell cycle arrest, apoptosis and autophagy. SIRT1 has recently been shown to be an important player in circadian regulation, which further intertwines the relationships among nutrient status, inflammation and cellular responses.

An interaction between inflammation and Sirt1 function has also been described. Acute phase inflammation, where TNFα expression is elevated, suppresses Sirt1 function and fatty acid oxidation (46, 67, 78, 83). There is also evidence that Sirt1 expression provides tolerance to TNFα and TLR responses, which supports the important relationships among metabolic regulation, inflammatory response and Sirt1 dependent functions (84–87). The hypothesis that TNFα is a direct regulator of Sirt expression was testing *in vivo* as well as in isolated hepatoctyes. We provide clear evidence that sirt expression in hepatocytes is regulated directly or indirectly through TNFα dependent signaling.

A major intracellular regulator of sirt1-dependent function recently described breast cancer cells but also in hepatocytes and other metabolic tissues is the protein Deleted in Breast Cancer 1 (DBC1) (11, 48, 49, 88, 89) DBC1 has been shown to directly bind SIRT1 and negatively regulate its activity as well as suppress sirt1 expression (47). Escande et al. showed that high fat diet increased DBC1 expression in mouse liver and that loss of *dbc1* protected mice from high fat diet induced fatty liver in a SIRT1 dependent fashion (48). A considerable amount of data now show that DBC1 works by binding and sequestering SIRT1, inhibiting its downstream function. Our experiments reveal an increase in DBC1 expression in wildtype mice with fatty liver that is significantly blunted in TNFR1-/- mice. Moreover, in isolated hepatocytes, TNFα directly increased DBC1 expression and interaction with SIRT1. Whether TNF induced DBC1:SIRT1 interaction accounts for the changes in hepatocyte lipid metabolism is not completely known but deserves further investigation.

This study highlights the association between inflammation and hepatic metabolic regulation *in vivo*. The important finding here is that the loss of TNF increases the capacity of the liver to utilize fatty acids for energy. It is demonstrated here that loss of TNF enhances Sirt1 expression and Sirt1-related adaptations in hepatic metabolism, suggesting that TNFα may be an important regulator of hepatic Sirt1 function possibly through DBC1 *in vivo* (**Figure 5E**). The TNF induced DBC1 and SIRT1 pathway may be an important pharmacological target to improve metabolic disorders, such as type 2 diabetes, fatty liver disease, and diseases with known associations between inflammation and metabolism.

## Data Availability

The raw data supporting the conclusions of this article will be made available by the authors, without undue reservation.

## References

[1] Tilg H Hotamisligil GS. Nonalcoholic fatty liver disease: Cytokine-adipokine interplay and regulation of insulin resistance. Gastroenterology. (2006) 131:934–45. doi: 10.1053/j.gastro.2006.05.054, PMID: 16952562

[2] Petrescu M Vlaicu SI Ciumărnean L Milaciu MV Mărginean C Florea M. Chronic inflammation – A link between nonalcoholic fatty liver disease (NAFLD) and dysfunctional adipose tissue. Medicina. (2022) 58:641. doi: 10.3390/medicina58050641, PMID: 35630058 PMC9147364

[3] Hotamisligil GS. Inflammation and metabolic disorders. Nature. (2006) 444:860–7. doi: 10.1038/nature05485, PMID: 17167474

[4] Lee YS Olefsky J. Chronic tissue inflammation and metabolic disease. Genes Dev. (2021) 35:307–28. doi: 10.1101/gad.346312.120, PMID: 33649162 PMC7919414

[5] Beutler B. Identity of tumour necrosis factor and the macrophage-secreted factor cachectin. Nature. (1985) 316:552–4. doi: 10.1038/316552a0, PMID: 2993897

[6] Beutler BA Milsark IW Cerami A. Cachectin/tumor necrosis factor: Production, distribution, and metabolic fate *in vivo* . J Immunol. (1985) 135:3972–7. doi: 10.4049/jimmunol.135.6.3972, PMID: 2999236

[7] Beutler B Grau GE. Tumor necrosis factor in the pathogenesis of infectious diseases. Crit Care Med. (1993) 21:S423–35. 8403980

[8] Hotamisligil GS Shargill NS Spiegelman BM. Adipose expression of tumor necrosis factor-alpha: Direct role in obesity-linked insulin resistance. Science. (1993) 259:87–91. doi: 10.1126/science.7678183, PMID: 7678183

[9] Alzamil H. Elevated serum TNF-α is related to obesity in type 2 diabetes mellitus and is associated with glycemic control and insulin resistance. J Obes. (2020) 2020:5076858. doi: 10.1155/2020/5076858, PMID: 32089876 PMC7013317

[10] Uysal KT Wiesbrock SM Marino MW Hotamisligil GS. Protection from obesity-induced insulin resistance in mice lacking TNF-alpha function. Nature. (1997) 389:610–4. doi: 10.1038/39335, PMID: 9335502

[11] Chen X Gong Q Wang CY Zhang K Ji X Chen YX. High-fat diet induces distinct metabolic response in interleukin-6 and tumor necrosis factor-α Knockout mice. J Interferon Cytokine Res. (2016) 36:580–8. doi: 10.1089/jir.2016.0022, PMID: 27610743

[12] Patsalos O Dalton B Leppanen J Ibrahim MAA Himmerich H. Impact of TNF-α Inhibitors on body weight and BMI: A systematic review and meta-analysis. Front Pharmacol. (2020) 11:481. doi: 10.3389/fphar.2020.00481, PMID: 32351392 PMC7174757

[13] Bugianesi E. Late complications of NASH: A challenge for hepatologists. J Hepatol. (2005) 42:784–5. doi: 10.1016/j.jhep.2005.02.007, PMID: 16649256

[14] Younossi ZM Henry L. Understanding the burden of nonalcoholic fatty liver disease: time for action. Diabetes Spectr. (2024) 37:9–19. doi: 10.2337/dsi23-0010, PMID: 38385101 PMC10877219

[15] Liu TF Vachharajani VT Yoza BK McCall CE. NAD^+^-dependent sirtuin 1 and 6 proteins coordinate a switch from glucose to fatty acid oxidation during the acute inflammatory response. J Biol Chem. (2012) 287:25758–69. doi: 10.1074/jbc.M112.362343, PMID: 22700961 PMC3406663

[16] Vachliotis ID Polyzos SA. The role of tumor necrosis factor-alpha in the pathogenesis and treatment of nonalcoholic fatty liver disease. Curr Obes Rep. (2023) 12:191–206. doi: 10.1007/s13679-023-00519-y, PMID: 37407724 PMC10482776

[17] Arkan MC. IKK-beta links inflammation to obesity-induced insulin resistance. Nat Med. (2005) 11:191–8. doi: 10.1038/nm1185, PMID: 15685170

[18] Tosello-Trampont AC Landes SG Nguyen V Novobrantseva TI Hahn YS. Kupffer cells trigger nonalcoholic steatohepatitis development in diet-induced mouse model through tumor necrosis factor-alpha production. J Biol Chem. (2012) 287:40161–72. doi: 10.1074/jbc.M112.417014, PMID: 23066023 PMC3504730

[19] Marra F Tacke F. Roles for chemokines in liver disease. Gastroenterology. (2014) 147:577–594.e1. doi: 10.1053/j.gastro.2014.06.043, PMID: 25066692

[20] Miura K Yang L van Rooijen N Ohnishi H Seki E. Hepatic recruitment of macrophages promotes nonalcoholic steatohepatitis through CCR2. Am J Physiol - Gastrointestinal Liver Physiol. (2012) 302:G1310–21. doi: 10.1152/ajpgi.00365.2011, PMID: 22442158 PMC3378163

[21] Baeck C Wehr A Karlmark KR Heymann F Vucur M Gassler N. Pharmacological inhibition of the chemokine CCL2 (MCP-1) diminishes liver macrophage infiltration and steatohepatitis in chronic hepatic injury. Gut. (2012) 61:416–26. doi: 10.1136/gutjnl-2011-300304, PMID: 21813474

[22] Obstfeld AE Sugaru E Thearle M Francisco AM Gayet C Ginsberg HN. C-C chemokine receptor 2 (CCR2) regulates the hepatic recruitment of myeloid cells that promote obesity-induced hepatic steatosis. Diabetes. (2010) 59:916–25. doi: 10.2337/db09-1403, PMID: 20103702 PMC2844839

[23] Li Z Yang S Lin H Huang J Watkins PA Moser AB. Probiotics and antibodies to TNF inhibit inflammatory activity and improve nonalcoholic fatty liver disease. Hepatology. (2003) 37:343–50. doi: 10.1053/jhep.2003.50048, PMID: 12540784

[24] Salles J Tardif N Landrier JF Mothe-Satney I Guillet C Boue-Vaysse C. TNFα Gene knockout differentially affects lipid deposition in liver and skeletal muscle of high-fat-diet mice. J Nutr Biochem. (2012) 23:1685–93. doi: 10.1016/j.jnutbio.2011.12.001, PMID: 22464148

[25] Schnyder-Candrian S Czarniecki J Lerondel S Corpataux J Ryffel B Schnyder B. Hepatic steatosis in the absence of tumor necrosis factor in mice. Cytokine. (2005) 32:287–95. doi: 10.1016/j.cyto.2005.11.004, PMID: 16406654

[26] Bluemel S Wang Y Lee S Schnabl B. Tumor necrosis factor alpha receptor 1 deficiency in hepatocytes does not protect from non-alcoholic steatohepatitis, but attenuates insulin resistance in mice. World J Gastroenterol. (2020) 26:4933–44. doi: 10.3748/wjg.v26.i33.4933, PMID: 32952340 PMC7476178

[27] Czaja MJ. JNK regulation of hepatic manifestations of the metabolic syndrome. Trends Endocrinol Metab. (2010) 21:707–13. doi: 10.1016/j.tem.2010.08.010, PMID: 20888782 PMC2991513

[28] Seki E Brenner DA Karin M. A liver full of JNK: Signaling in regulation of cell function and disease pathogenesis, and clinical approaches. Gastroenterology. (2012) 143:307–20. doi: 10.1053/j.gastro.2012.06.004, PMID: 22705006 PMC3523093

[29] Wandrer F Liebig S Marhenke S Vogel A John K Manns MP. TNF-receptor-1 inhibition reduces liver steatosis, hepatocellular injury and fibrosis in NAFLD mice. Cell Death Dis. (2020) 11:212. doi: 10.1038/s41419-020-2411-6, PMID: 32235829 PMC7109108

[30] Wu T Liu YH Fu YC Liu XM Zhou XH. Direct evidence of sirtuin downregulation in the liver of non-alcoholic fatty liver disease patients. Ann Clin Lab Sci. (2014) 44:410–8., PMID: 25361925

[31] Yin H Hu M Liang X Ajmo JM Li X Bataller R. Deletion of SIRT1 from hepatocytes in mice disrupts lipin-1 signaling and aggravates alcoholic fatty liver. Gastroenterology. (2014) 146:801–11. doi: 10.1053/j.gastro.2013.11.008, PMID: 24262277 PMC3943758

[32] Ding RB Bao J Deng CX. Emerging roles of SIRT1 in fatty liver diseases. Int J Biol Sci. (2017) 13:852–67. doi: 10.7150/ijbs.19370, PMID: 28808418 PMC5555103

[33] Picard F Kurtev M Chung N Topark-Ngarm A Senawong T Machado De Oliveira R. Sirt1 promotes fat mobilization in white adipocytes by repressing PPAR-gamma. Nature. (2004) 429:771–6. doi: 10.1038/nature02583, PMID: 15175761 PMC2820247

[34] Rodgers JT Lerin C Haas W Gygi SP Spiegelman BM Puigserver P. Nutrient control of glucose homeostasis through a complex of PGC-1alpha and SIRT1. Nature. (2005) 434:113–8. doi: 10.1038/nature03354, PMID: 15744310

[35] Xu F Gao Z Zhang J Rivera CA Yin J Weng J. Lack of SIRT1 (mammalian sirtuin 1) activity leads to liver steatosis in the SIRT1+/- mice: A role of lipid mobilization and inflammation. Endocrinology. (2010) 151:2504–14. doi: 10.1210/en.2009-1013, PMID: 20339025 PMC2875813

[36] Zeng C Chen M. Progress in nonalcoholic fatty liver disease: SIRT family regulates mitochondrial biogenesis. Biomolecules. (2022) 12:1079. doi: 10.3390/biom12081079, PMID: 36008973 PMC9405760

[37] Purushotham A Xu Q Lu J Foley JF Yan X Kim DH. Hepatic deletion of SIRT1 decreases hepatocyte nuclear factor 1alpha/farnesoid X receptor signaling and induces formation of cholesterol gallstones in mice. Mol Cell Biol. (2012) 32:1226–36. doi: 10.1128/MCB.05988-11, PMID: 22290433 PMC3302441

[38] Reinke LA Thurman RG Kauffman FC. Oxidation-reduction state of free NADP+ during mixed-function oxidation in perfused rat livers—evaluation of the assumptions of near equilibrium by comparisons of surface fluorescence changes and calculated NADP+:NADPH ratios. Biochem Pharmacol. (1979) 28:2381–7. doi: 10.1016/0006-2952(79)90704-4, PMID: 40558

[39] Kremer M Thomas E Milton RJ Perry AW van Rooijen N Wheeler MD. Kupffer cell and interleukin-12-dependent loss of natural killer T cells in hepatosteatosis. Hepatology. (2010) 51:130–41. doi: 10.1002/hep.23292, PMID: 20034047 PMC3761962

[40] Isayama F Hines IN Kremer M Milton RJ Byrd CL Perry AW. LPS signaling enhances hepatic fibrogenesis caused by experimental cholestasis in mice. Am J Physiol - Gastrointestinal Liver Physiol. (2006) 290:G1318–28. doi: 10.1152/ajpgi.00405.2005, PMID: 16439470

[41] Qu W Graves LM Thurman RG. PGE2 stimulates O2 uptake in hepatic parenchymal cells: Involvement of the cAMP-dependent protein kinase. Am J Physiol. (1999) 277:G1048–54. doi: 10.1152/ajpgi.1999.277.5.G1048, PMID: 10564111

[42] Nakamura T Nakamura S Onodera A Suzuki O Aikawa T Karoji N. Effects of Alcohol and Nutrition on Hepatic Lipids in Rats. Tohoku J Exp Med. (1967) 93:227–233., PMID: 5626540 10.1620/tjem.93.227

[43] Ross A Caballero B Cousins R Tucker K Ziegler T, editors. Modern Nutrition in Health and Disease, 11th ed. Philadelphia: Wolters Kluwer Health/Lippincott Williams & Wilkins (2014) 416–26.

[44] Guarente L Picard F. Calorie restriction—the SIR2 connection. Cell. (2005) 120:473–82. doi: 10.1016/j.cell.2005.01.029, PMID: 15734680

[45] Motta MC. Mammalian SIRT1 represses forkhead transcription factors. Cell. (2004) 116:551–63. doi: 10.1016/S0092-8674(04)00126-6, PMID: 14980222

[46] Yang Y Liu Y Wang Y Chao Y Zhang J Jia Y. Regulation of SIRT1 and its roles in inflammation. Front Immunol. (2022) 13:831168. doi: 10.3389/fimmu.2022.831168, PMID: 35359990 PMC8962665

[47] Kim HJ Moon SJ Kim JH. Mechanistic insights into the dual role of CCAR2/DBC1 in cancer. Exp Mol Med. (2023) 55:1691–701. doi: 10.1038/s12276-023-01058-1, PMID: 37524873 PMC10474295

[48] Escande C Chini CC Nin V Dykhouse KM Novak CM Levine J. Deleted in breast cancer-1 regulates SIRT1 activity and contributes to high-fat diet-induced liver steatosis in mice. J Clin Invest. (2010) 120:545–58. doi: 10.1172/JCI39319, PMID: 20071779 PMC2810074

[49] Nin V Escande C Chini CC Giri S Camacho-Pereira J Matalonga J. Role of deleted in breast cancer 1 (DBC1) protein in SIRT1 deacetylase activation induced by protein kinase A and AMP-activated protein kinase. J Biol Chem. (2012) 287:23489–501. doi: 10.1074/jbc.M112.365874, PMID: 22553202 PMC3390625

[50] Cai D Yuan M Frantz DF Melendez PA Hansen L Lee J. Local and systemic insulin resistance resulting from hepatic activation of IKK-beta and NF-kappaB. Nat Med. (2005) 11:183–90. doi: 10.1038/nm1166, PMID: 15685173 PMC1440292

[51] Cave M Deaciuc I Mendez C Song Z Joshi-Barve S Barve S. Nonalcoholic fatty liver disease: Predisposing factors and the role of nutrition. J Nutr Biochem. (2007) 18:184–95. doi: 10.1016/j.jnutbio.2006.12.006, PMID: 17296492

[52] Feldstein AE Canbay A Guicciardi ME Higuchi H Bronk SF Gores GJ. Diet-associated hepatic steatosis sensitizes to Fas-mediated liver injury in mice. J Hepatol. (2003) 39:978–83. doi: 10.1016/S0168-8278(03)00460-4, PMID: 14642615

[53] Pan X Chiwanda Kaminga A Liu A Wen SW Chen J Luo J. Chemokines in non-alcoholic fatty liver disease: A systematic review and network meta-analysis. Front Immunol. (2020) 11:1802. doi: 10.3389/fimmu.2020.01802, PMID: 33042108 PMC7530185

[54] Brun P Castagliuolo I Di Leo V Buda A Pinzani M Palù G. Increased intestinal permeability in obese mice: New evidence in the pathogenesis of nonalcoholic steatohepatitis. Am J Physiol - Gastrointestinal Liver Physiol. (2007) 292:G518–25. doi: 10.1152/ajpgi.00024.2006, PMID: 17023554

[55] Lee JY Plakidas A Lee WH Heikkinen A Chanmugam P Bray G. Differential modulation of toll-like receptors by fatty acids: Preferential inhibition by n-3 polyunsaturated fatty acids. J Lipid Res. (2003) 44:479–86. doi: 10.1194/jlr.M200361-JLR200, PMID: 12562875

[56] Rivera CA Adegboyega P van Rooijen N Tagalicud A Allman M Wallace M. Toll-like receptor-4 signaling and Kupffer cells play pivotal roles in the pathogenesis of non-alcoholic steatohepatitis. J Hepatol. (2007) 47:571–9. doi: 10.1016/j.jhep.2007.04.019, PMID: 17644211 PMC2094119

[57] Hines IN Milton J Kremer M Wheeler MD. Ablation of tumor necrosis factor alpha receptor 1 signaling blunts steatohepatitis in peroxisome proliferator activated receptor α-deficient mice. Med Res Arch. (2022) 10:3082. doi: 10.18103/mra.v10i9.3082, PMID: 36865784 PMC9977327

[58] Dela Peña A Leclercq I Field J George J Jones B Farrell G. NF-kappaB activation, rather than TNF, mediates hepatic inflammation in a murine dietary model of steatohepatitis. Gastroenterology. (2005) 129:1663–74. doi: 10.1053/j.gastro.2005.09.004, PMID: 16285964

[59] Deng QG She H Cheng JH French SW Koop DR Xiong S. Steatohepatitis induced by intragastric overfeeding in mice. Hepatology. (2005) 42:905–14. doi: 10.1002/hep.20877, PMID: 16175602

[60] García-Ruiz I Rodríguez-Juan C Díaz-Sanjuan T del Hoyo P Colina F Muñoz-Yagüe T. Uric acid and anti-TNF antibody improve mitochondrial dysfunction in ob/ob mice. Hepatology. (2006) 44:581–91. doi: 10.1002/hep.21313, PMID: 16941682

[61] Memon RA Grunfeld C Feingold KR. TNF-alpha is not the cause of fatty liver disease in obese diabetic mice. Nat Med. (2001) 7:2–3. doi: 10.1038/83316, PMID: 11135585

[62] Rafaqat S Gluscevic S Mercantepe F Rafaqat S Klisic A. Interleukins: pathogenesis in non-alcoholic fatty liver disease. Metabolites. (2024) 14:153. doi: 10.3390/metabo14030153, PMID: 38535313 PMC10972081

[63] Tilg H Moschen AR. IL-1 cytokine family members and NAFLD: neglected in metabolic liver inflammation. J Hepatol. (2011) 55:960–2. doi: 10.1016/j.jhep.2011.04.00, PMID: 21742000

[64] Kamari Y Shaish A Vax E Shemesh S Kandel-Kfir M Arbel Y. Lack of interleukin-1α or interleukin-1β Inhibits transformation of steatosis to steatohepatitis and liver fibrosis in hypercholesterolemic mice. J Hepatol. (2011) 55:1086–94. doi: 10.1016/j.jhep.2011.01.048, PMID: 21354232 PMC3210940

[65] Gieling RG Wallace K Han YP. Interleukin-1 participates in the progression from liver injury to fibrosis. Am J Physiol - Gastrointestinal Liver Physiol. (2009) 296:G1324–31. doi: 10.1152/ajpgi.90564.2008, PMID: 19342509 PMC2697947

[66] Ventre J Doebber T Wu M MacNaul K Stevens K Pasparakis M. Targeted disruption of the tumor necrosis factor-alpha gene: Metabolic consequences in obese and nonobese mice. Diabetes. (1997) 46:1526–31. doi: 10.2337/diab.46.9.1526, PMID: 9287059

[67] Perry RJ Camporez JG Kursawe R. Hepatic acetyl coA links adipose tissue inflammation to hepatic insulin resistance and type 2 diabetes. Cell. (2015) 160:745–58. doi: 10.1016/j.cell.2015.01.012, PMID: 25662011 PMC4498261

[68] Noga AA Vance DE. A gender-specific role for phosphatidylethanolamine N-methyltransferase-derived phosphatidylcholine in the regulation of plasma high density and very low density lipoproteins in mice. J Biol Chem. (2003) 278:21851–9. doi: 10.1074/jbc.M301982200, PMID: 12668679

[69] Li Z Vance DE. Phosphatidylcholine and choline homeostasis. J Lipid Res. (2008) 49:1187–94. doi: 10.1194/jlr.R700019-JLR200, PMID: 18204095

[70] Corbin KD Zeisel SH. Choline metabolism provides novel insights into nonalcoholic fatty liver disease and its progression. Curr Opin. (2012) 28:159–65. doi: 10.1097/MOG.0b013e32834e7b4b, PMID: 22134222 PMC3601486

[71] Ishigure T Sasase T Tohma M Uno K Toriniwa Y Saito T. Choline-deficient diet-induced NAFLD animal model recaptures core human pathophysiology with similar gene co-expression networks. In Vivo. (2023) 37:pp.1517–1531. doi: 10.21873/invivo.13237, PMID: 37369510 PMC10347920

[72] Marchesini G Marzocchi R Agostini F Bugianesi E. Nonalcoholic fatty liver disease and the metabolic syndrome. Curr Opin Lipidology. (2005) 16:421–7. doi: 10.1097/01.mol.0000177191.01612.85 15990591

[73] Svegliati-Baroni G Bugianesi E Bouserhal T Marini F Ridolfi F Tarsetti F. Post-load insulin resistance is an independent predictor of hepatic fibrosis in virus C chronic hepatitis and in non-alcoholic fatty liver disease. Gut. (2007) 56:1296–301. doi: 10.1136/gut.2006.107946, PMID: 17392334 PMC1954981

[74] Kralj D Virović Jukić L Stojsavljević S Duvnjak M Smolić M Čurčić IB. Hepatitis C virus, insulin resistance, and steatosis. J Clin Trans Hepatol. (2016) 4:66–75. doi: 10.14218/JCTH.2015.00051, PMID: 27047774 PMC4807145

[75] Kim MS Sweeney TR Shigenaga JK Chui LG Moser A Grunfeld C. Tumor necrosis factor and interleukin 1 decrease RXRalpha, PPARalpha, PPARgamma, LXRalpha, and the coactivators SRC-1, PGC-1alpha, and PGC-1beta in liver cells. Metabolism. (2007) 56:267–79. doi: 10.1016/j.metabol.2006.09.008, PMID: 17224343 PMC2700944

[76] Chen X Li D Yang Y et al. Tumor necrosis factor α promotes hyperlipidemia and hepatic lipid accumulation through downregulation of peroxisome proliferator–activated receptor α expression. J Lipid Res. (2009) 50:2335–2343. doi: 10.1194/jlr.M800657-JLR200, PMID: 19395734 PMC2724773

[77] Beier K Völkl A Fahimi HD. Suppression of peroxisomal lipid beta-oxidation enzymes of TNF-alpha. FEBS Lett. (1992) 310:273–6. doi: 10.1016/0014-5793(92)81347-O, PMID: 1397283

[78] Chen X Xun K Chen L Wang Y. TNF-alpha, a potent lipid metabolism regulator. Cell Biochem Funct. (2009) 27:407–16. doi: 10.1002/cbf.1596, PMID: 19757404

[79] Li X Zhang S Blander G Tse JG Krieger M Guarente L. SIRT1 deacetylates and positively regulates the nuclear receptor LXR. Mol Cell. (2007) 28:91–106. doi: 10.1016/j.molcel.2007.07.032, PMID: 17936707

[80] Rodgers JT Puigserver P. Fasting-dependent glucose and lipid metabolic response through hepatic sirtuin 1. Proc Natl Acad Sci United States America. (2007) 104:12861–6. doi: 10.1073/pnas.0702509104, PMID: 17646659 PMC1937557

[81] Bordone L Cohen D Robinson A Motta MC van Veen E Czopik A. SIRT1 transgenic mice show phenotypes resembling calorie restriction. Aging Cell. (2007) 6:759–67. doi: 10.1111/j.1474-9726.2007.00335.x, PMID: 17877786

[82] Purushotham A Schug TT Xu Q Surapureddi S Guo X Li X. Hepatocyte-specific deletion of SIRT1 alters fatty acid metabolism and results in hepatic steatosis and inflammation. Cell Metab. (2009) 9:327–38. doi: 10.1016/j.cmet.2009.02.006, PMID: 19356714 PMC2668535

[83] Liu TF Brown CM El Gazzar M McPhail L Millet P Rao A. Fueling the flame: Bioenergy couples metabolism and inflammation. J Leukocyte Biol. (2012b) 92:499–507. doi: 10.1189/jlb.0212078, PMID: 22571857 PMC3427613

[84] Yeung F Hoberg JE Ramsey CS Keller MD Jones DR Frye RA. Modulation of NF-κB-dependent transcription and cell survival by the SIRT1 deacetylase. EMBO J. (2004) 23:2369–80. doi: 10.1038/sj.emboj.7600244, PMID: 15152190 PMC423286

[85] Liu TF Yoza BK El Gazzar M Vachharajani VT McCall CE. NAD^+^-dependent SIRT1 deacetylase participates in epigenetic reprogramming during endotoxin tolerance. J Biol Chem. (2011) 286:9856–64. doi: 10.1074/jbc.M110.196790, PMID: 21245135 PMC3058977

[86] de Gregorio E Colell A Morales A Marí M. Relevance of SIRT1-NF-κB axis as therapeutic target to ameliorate inflammation in liver disease. Int J Mol Sci. (2020) 21:3858. doi: 10.3390/ijms21113858, PMID: 32485811 PMC7312021

[87] Fortuny L Sebastián C. Sirtuins as metabolic regulators of immune cells phenotype and function. Genes (Basel). (2021) 12:1698. doi: 10.3390/genes12111698, PMID: 34828304 PMC8618532

[88] Kim JE Chen J Lou Z. DBC1 is a negative regulator of SIRT1. Nature. (2008) 451:583–6. doi: 10.1038/nature06500, PMID: 18235501

[89] Zhao W Kruse JP Tang Y Jung SY Qin J Gu W. Negative regulation of the deacetylase SIRT1 by DBC1. Nature. (2008) 451:587–90. doi: 10.1038/nature06515, PMID: 18235502 PMC2866287

