# Supplementary figures and images for: Regulation of hepatic Sirt1 expression and lipid metabolism through TNF receptor signaling
Source: Front Immunol. 2025 Jul 22;16:1627433. doi: 10.3389/fimmu.2025.1627433 (PMC12321515; doi:10.3389/fimmu.2025.1627433)

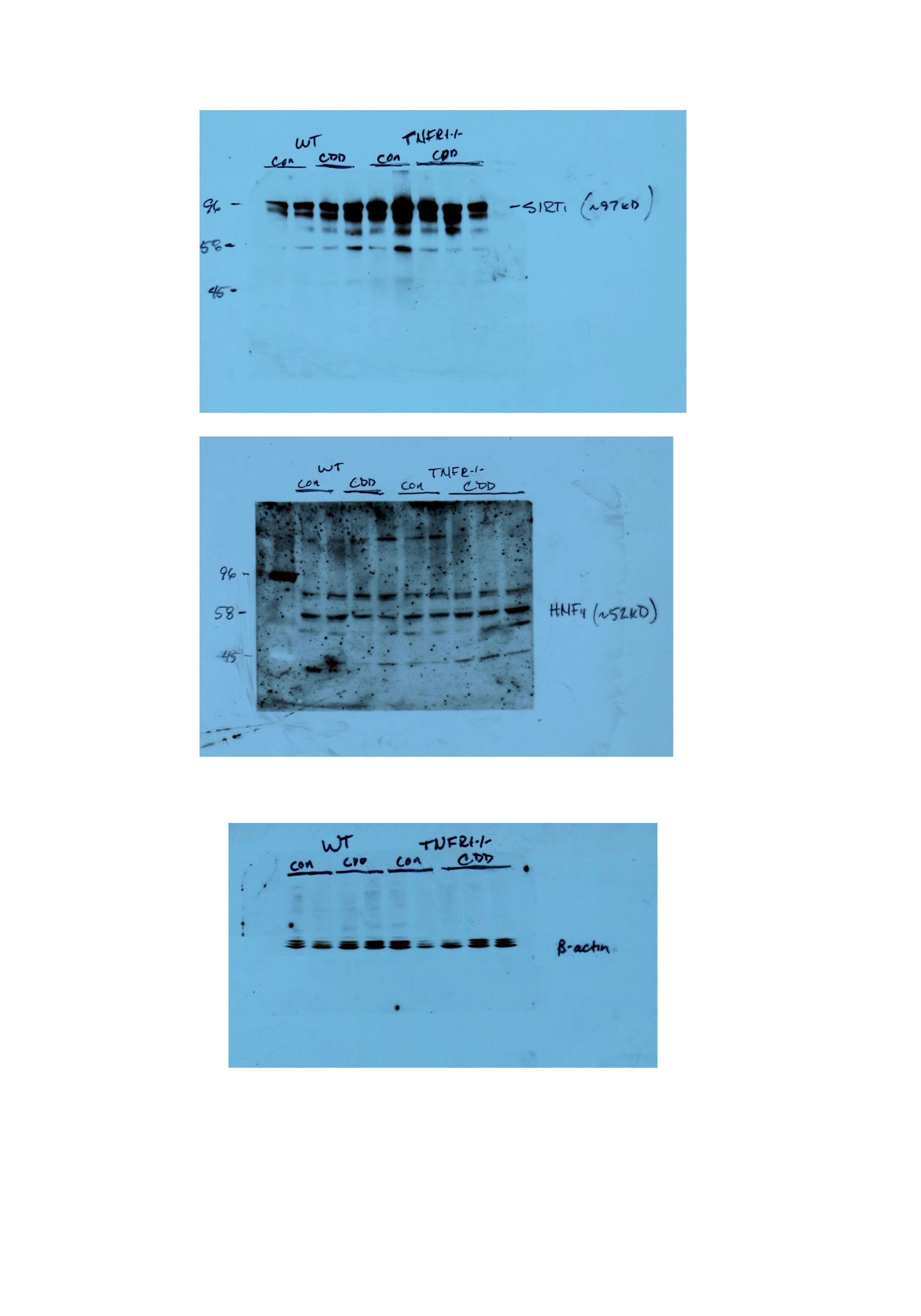

Supplement: Supplementary file 1 [file Image1.jpeg]
